# Medication Possession Ratio Predicts Antiretroviral Regimens Persistence in Peru

**DOI:** 10.1371/journal.pone.0076323

**Published:** 2013-10-01

**Authors:** Jorge L. Salinas, Jorge L. Alave, Andrew O. Westfall, Jorge Paz, Fiorella Moran, Danny Carbajal-Gonzalez, David Callacondo, Odalie Avalos, Martin Rodriguez, Eduardo Gotuzzo, Juan Echevarria, James H. Willig

**Affiliations:** 1 Department of Medicine, University of Alabama at Birmingham, Birmingham, Alabama, United States of America; 2 Hospital Nacional Cayetano Heredia, Ministerio de Salud, Lima, Peru; 3 Department of Biostatistics, University of Alabama at Birmingham, Birmingham, Alabama, United States of America; 4 Instituto de Medicina Tropical Alexander von Humboldt, Universidad Peruana Cayetano Heredia, Lima, Peru; 5 Servicio Rural y Urbano Marginal de Salud, Ministerio de Salud, Lima, Peru; 6 Laboratorios de Investigacion y Desarrollo, Department of Microbiology, Universidad Peruana Cayetano Heredia, Lima, Peru; National HIV and Retrovirology Laboratories, Canada

## Abstract

**Objectives:**

In developing nations, the use of operational parameters (OPs) in the prediction of clinical care represents a missed opportunity to enhance the care process. We modeled the impact of multiple measurements of antiretroviral treatment (ART) adherence on antiretroviral treatment outcomes in Peru.

**Design And Methods:**

Retrospective cohort study including ART naïve, non-pregnant, adults initiating therapy at Hospital Nacional Cayetano Heredia, Lima-Peru (2006-2010). Three OPs were defined: 1) Medication possession ratio (MPR): days with antiretrovirals dispensed/days on first-line therapy; 2) Laboratory monitory constancy (LMC): proportion of 6 months intervals with ≥1 viral load or CD4 reported; 3) Clinic visit constancy (CVC): proportion of 6 months intervals with ≥1 clinic visit.

Three multi-variable Cox proportional hazard (PH) models (one per OP) were fit for (1) time of first-line ART persistence and (2) time to second-line virologic failure. All models were adjusted for socio-demographic, clinical and laboratory variables.

**Results:**

856 patients were included in first-line persistence analyses, median age was 35.6 years [29.4-42.9] and most were male (624; 73%). In multivariable PH models, MPR (per 10% increase HR=0.66; 95%CI=0.61-0.71) and LMC (per 10% increase 0.83; 0.71-0.96) were associated with prolonged time on first-line therapies.

Among 79 individuals included in time to second-line virologic failure analyses, MPR was the only OP independently associated with prolonged time to second-line virologic failure (per 10% increase 0.88; 0.77-0.99).

**Conclusions:**

The capture and utilization of program level parameters such as MPR can provide valuable insight into patient-level treatment outcomes.

## Introduction

HIV treatment programs have been established throughout the developing world resulting in rapid expansion of the number of individuals receiving therapy and significant decrements in HIV related morbidity and mortality [1]. Presently, many of these programs face limited numbers of available first-line drugs, costly second-line regimens [2], and little to no access to third-line regimens. Because first-line regimens are the longest lasting regimens, when not affected by toxicity, maximizing their persistence or durability becomes a key component of the strategy for long-term programmatic success [3-5].

In the last decade, several operational parameters (OPs) such as medication possession ratio (MPR), laboratory monitoring constancy (LMC) and clinic visit constancy (CVC) have emerged as objective methods of measuring retention in HIV care and have been shown to be good predictors of important clinical outcomes including HIV drug resistance [2,6,7], virologic failure [8,9] and mortality [4,10]. Despite their potential utility, not all OPs are systematically captured in health system monitoring, making their use non-standardized, difficult or even not feasible in resource-limited settings (RLS). In addition, head to head comparisons among the different OPs and their relation to patient-level outcomes are scarce, and in particular the relationship between such parameters and antiretroviral (ART) regimen persistence remains understudied [4,6,7,10,11]. In the present investigation, we assess the utility of three OPs (MPR, CVC and LMC) in examining the correlates of first-line ART regimen persistence and second regimen virologic failure in Peru, a middle-income country.

## Methods

### Ethics Statement

All patients enrolled in the Hospital Nacional Cayetano Heredia (HNCH) HIV cohort sign an informed consent allowing the use of their data in clinical research. We used de-identified data previously recorded in the HNCH HIV cohort; as such patients were not contacted for this particular study. Institutional review boards at HNCH and the University of Alabama at Birmingham (UAB) approved this study.

### Study Setting and Design

The HNCH is part of the Peruvian National HIV program and includes a cohort of over 1600 patients receiving HIV/AIDS care. This prospective cohort collects detailed socio-demographic, psychosocial, and clinical information on all patients enrolled in care. All HIV care is provided on-site, and is coordinated by a team of infectious disease specialists. For patients prescribed ART during the observation period from January 2006 through December 2010 (CD4<200 cells/ml or symptomatic), patients are scheduled for visits in three concurrent facets of the system: medication pick-up, laboratory appointments and clinic visits. Medications are dispensed free of charge, directly to patients by a team of nurses. Initially, however, a 1-week supply of ART supply is dispensed for the first 2 weeks of therapy and monthly supplies are dispensed thereafter if there are no complications. Patients are scheduled for separate laboratory monitoring (CD4 count and viral load) visits every six months (HIV genotyping is not routinely obtained). Patients also attend clinic visits for evaluation by an infectious disease provider at least once every six months. Both laboratory testing and clinic visit frequency take place as recommended by the Peruvian Ministry of Health guidelines [12]. Due to operational constraints such as the lack of coordination across environments and staffing limitations, medication pick-up, laboratory and clinic appointments often does not occur on the same date. All laboratory and clinical information is stored in an electronic database on site at the HNCH.

### Eligibility Criteria

All treatment-naive patients 18 years or older and initiating ART between January 2, 2006 and December 30, 2010 were included. Treatment initiation was defined as the start of a first-line regimen (2 nucleoside reverse transcriptase inhibitors (NRTI) and 1 non nucleoside reverse transcriptase inhibitor (NNRTI)) [13] and continuation for ≥ 14 days.

Patients who discontinue ART during the first two weeks primarily do so for adverse side effects and were excluded from the analysis. Also excluded were pregnant women (As this patients normally switch to PIs containing ART regimens due to pregnancy and not due to failure), patients whose initial regimens included a protease inhibitor (PI; therefore non-first-line regimen per Peruvian National Guidelines) and patients purchasing their medications privately (hence not receiving standard issue regimens dispensed via the national treatment program).

### Study variables

Socio-demographic, psychosocial, and clinical variables were retrieved by direct chart abstraction from clinic notes (J.L.S., J.L.A., J.P., F.M., D.C.G., D.C.), medication pick up details were retrieved from standardized ART dispensing documents while laboratory data and clinic visit dates were extracted via Microsoft Access queries to the HNCH database where all arrived laboratory visit dates are recorded. A number of independent patient-level variables included age, gender, baseline weight (kg), HIV acquisition risk factor (heterosexual, men who have sex with men [MSM]) and standardized body mass index (BMI): weight/(height in meters)^2^. Socio-economic variables included travel distance from home district to hospital (in miles, using Google Maps: maps.google.com consulted on February 2011), and income (obtained from social worker assessments prior to ART initiation where poverty was stratified as earning < minimum wage and extreme poverty: < half the minimum wage) [14]. Clinical data included medical history of opportunistic infections, tuberculosis and sexually transmitted diseases (STD) prior to ART initiation. Laboratory variables included baseline and subsequent values for the following tests: HIV RNA (copies/ml), CD4 value (cells/ml), and hematocrit (%). Antiretroviral regimen variables were collected for both first-line regimens (defined as 2 NRTIs and an NNRTI) and second-line regimens (NRTIs in combination with a PI), if prescribed. We also constructed 2 clinical markers, CD4 and BMI progression, comparing baseline values with values at the beginning of second-line regimens: (1) CD4 progression: A positive progression was dichotomously defined as either achieving a CD4 count above 200 cells/ml in those starting with a CD4 < 200 or having a CD4 value higher than baseline at the end of the first-line in those starting with CD4 ≥ 200 (2) BMI progression: was dichotomously stratified as positive if there was any increase of BMI noted, otherwise was recorded as negative.

Three OPs, as previously validated, were constructed by the study statistician using pharmacy pick-up, laboratory completion and clinic arrived visit dates; OPs were measured only during the first-line period. They were:

1 Medication possession ratio (MPR): days with antiretrovirals dispensed/days on first-line therapy [15];
2 Laboratory monitory constancy (LMC): proportion of 6 months intervals in follow-up with ≥ 1 viral load (VL) or CD4 reported [16];
3 Clinic visit constancy (CVC): proportion of 6 months intervals in follow-up with ≥ 1 clinic visit [4,17].

### Dependent variables

Two primary study outcomes were decided *a priori* and included:

1 First-line regimen persistence was measured as the time from first-line regimen initiation to switch to a second-line regimen (NRTIs and a PI) or to discontinuation of medications completely. Patients who had not discontinued their first-line regimen were censored at date of death, date lost to follow-up (30 days past expected medication run out date per drug pick-up data, as recommended by the Peruvian national guidelines), date of transfer of care or administratively at January 31^st^, 2011 (end of observation period).
2 Time to virologic failure of a second-line regimen, measured from second-line initiation (defined as start of a PI containing regimen) to the first viral load ≥ 400 copies/ml after 90 days of therapy. Patients who started a second-line regimen and had at least 1 follow-up viral load more than 90 days after the start of a second-line regimen were included. Patients who did not experience virologic failure were censored at the date of the last available viral load.

The lack of third-line drugs in Peru at the time these data were captured made the measurement of second-line regimen persistence impossible. We chose time to virologic failure as a surrogate of second-line persistence since it would be a reasonable indication to the time to switch to third-line regimens if these were available. We used 90 days instead of the more conventional 180 days as the cut-off for virologic failure as most patients lacked VL measured after 180 days, this definition has been used in other studies in the literature [18,19].

### Statistical analysis

Descriptive statistics were employed to evaluate overall patient and regimen-level characteristics to ensure distributional assumptions for statistical tests were met. Continuous variables are reported as median [first quartile-third quartile] and categorical variables are reported as frequencies and percentages. Univariate analyses (UV) were performed to identify factors affecting initial regimen longevity and time to second-line regimen failure. Next, three multi-variable proportional hazard (PH) models (one per each OP) were fit for each of our study outcomes:

1 Time to second-line ART initiation;
2 Time to virologic failure of second-line ART.

Each model was adjusted for socio-demographic, clinical and laboratory variables. OPs were treated as time-varying covariates for first-line persistence assessments but fixed for second-line regimen time to virologic failure. We carried out sensitivity analyses using MPR as a continuous and as a categorical variable (using 85% as the cut off), both were time-varying measures over the preceding 180 days; thus, Hazard Ratios (HR) for these two analyses can be interpreted at any given time during the first-line period (≥180 days). Analyses were performed using SAS V9.1.3 software (SAS Institute, Cary, NC).

## Results

**Figure 1:**
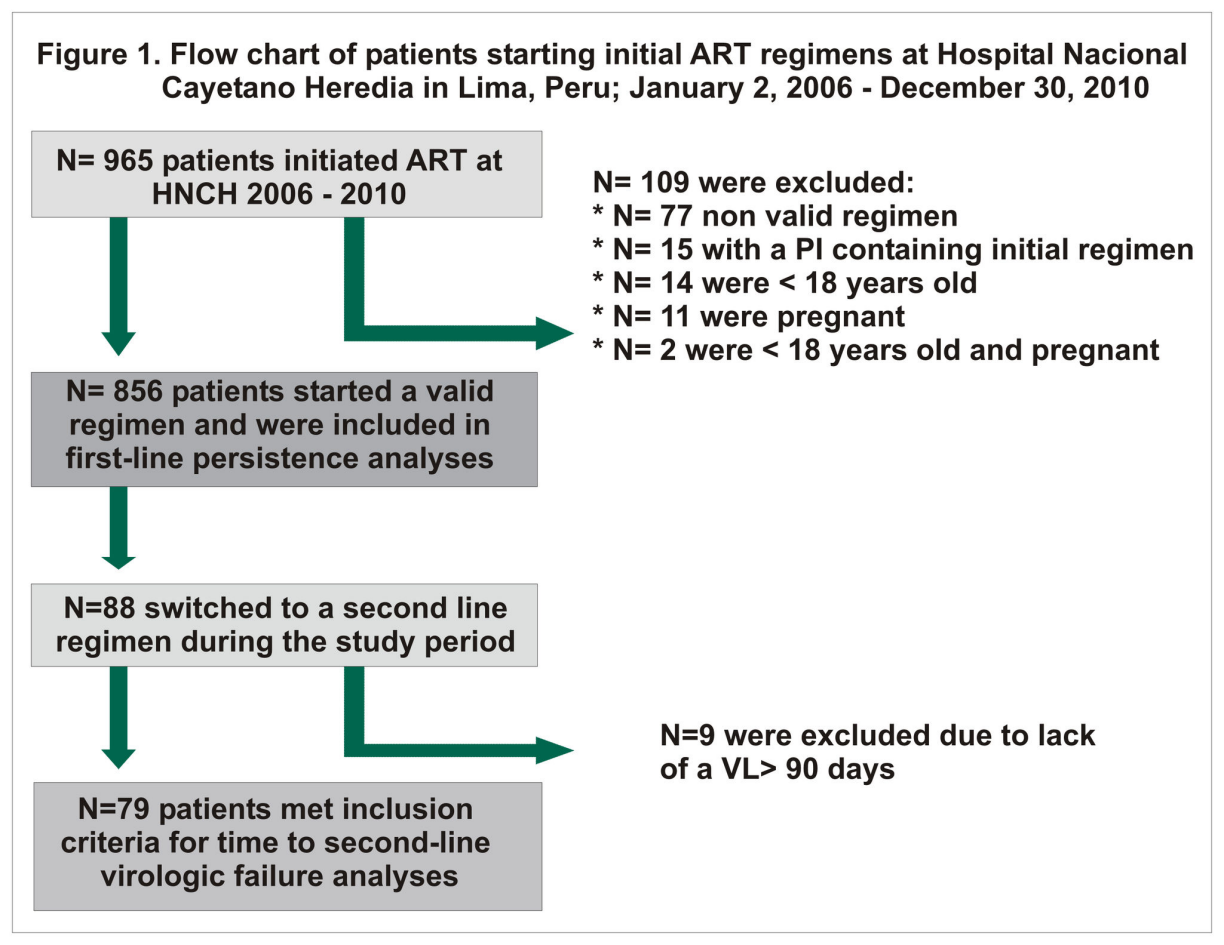
Flow chart of patients starting initial ART regimens at Hospital Nacional Cayetano Heredia in Lima, Peru; January 2, 2006-December 30,2010.

From January 2006 to December 2009; 965 patients started initial ART at HNCH in Lima Peru; 109 individuals did not meet study entry criteria (Figure 1) and 856 patients began a valid first-line regimen (see table 1 for baseline characteristics). A total of 88 patients switched to second-line regimens; of those, 9 individuals were excluded due to lack of a VL > 90 days from switch date and the remaining 79 patients were included in the analysis of second-line regimens and virologic failure.

**Table 1:** Baseline characteristics of antiretroviral-naive patients starting initial ART regimens and patients switching to a second line regimen at Hospital Nacional Cayetano Heredia in Lima, Peru; January 2, 2006-December 30, 2010.

	**First line persistence (N=856**)	**Time to virologic failure on 2nd line (N=79)**
**Characteristics**	**Median (IQR) or N (%)**	
Age	35.6 [29.4-42.9]	35.7 [27.9-42.6]
Sex		
Female	232 (27.1%)	19 (24.1%)
Baseline weight (kg)	59 [52-66]	57 [48.5-63.5]
Income		
Living in extreme poverty	98 (15.9%)	11 (18%)
Travel distance from home to hospital	5.2 [2.2-12.1]	5.2 [3.6-12.5]
History opportunistic infection		
Yes	684 (79.9%)	68 (86.1%)
History of prior TB infection		
Yes	177 (20.7%)	21 (26.6)
History of STD		
Yes	218 (25.5%)	23 (29.1%)
Baseline CD4 (cells/ml)	95 [42-200]	76 [30-155]
Baseline viral load (VL) (x1000 copies/ml)	124.7 [35.8-315]	123.2 [36.6-275.3]
Baseline hematocrit (HCY) (mg/dl)	36 [32-40]	36 [31.1-38]
Duration of first line (days)*	-	413 [192-753]
Prior ARV exposure *		
3	-	70 (88.6%)
4 or 5	-	9 (11.4%)
New drugs in initial 2nd-line regimen&*		
1	-	39 (49.4%)
2 or 3	-	40 (50.6%)
Closest VL to second line initiation	-	38.4 [4.4-207.4]
CD4 progression over first line*		
Positive	-	19 (36.5%)
Negative	-	33 (63.5%)
BMI Progression over first line *		
Positive	-	46 (70.8%)
Negative	-	19 (29.2%)

Abbreviations: TB, tuberculosis; STD, sexually transmitted diseases; ARV, antiretroviral; BMI, body mass index.

¶ Missing Values: weight: (part A=73, part B=3); Income: (part A=239, part B=18); Travel distance: (part A=172, part B=16); Baseline CD4: (part A=52, part B=6); Baseline VL: (part A=196, part B=19); CD4 progression over first line=27; Closest VL to Second Line Initiation=14; BMI progression over first line=14.

* Characteristics only recorded at the beginning of the second line regimens.

& Zidovudine and stavudine are counted as the same ARV given their similar resistance patterns.

### First-line persistence analyses

We included 856 patients in first-line regimen persistence analyses. Among these, age was 35.6 years [interquartile range (IQR) 29.4-42.9]; most were male (n=624, 73%) and baseline CD4 was 95 cells/ml (IQR 42-200) (Table 1). In UV analysis, MPR (per 10% increase) (HR=0.69; 95% confidence interval=0.60-0.70) and LMC (per 10% increase) (HR=0.88; 0.70-0.90) were associated with prolonged first-line persistence while CVC was not (HR=0.94; 0.84-1.04). Baseline CD4 count (per 50 cells/ml) was the only laboratory variable value associated with a decreased hazard of first-line regimen discontinuation (HR=0.87; 0.77-0.97). All other demographic and laboratory variables did not meet statistical significance.

For first-line regimen persistence we fit three multivariable proportional hazard models, one for each OP (Table 2). The first model, included MPR, showed that 10% increments in MPR (HR=0.66; 0.61-0.71) were associated with decreased hazard of switch to a second-line regimens while demographic, clinical or laboratory characteristics failed to show an association with prolonged time on first-line regimens. We then performed sensitivity analyses using MPR. When measured as a continuous variable over the preceding 180 days, 10% increases in MPR were associated with a decreased risk of switching to a second-line regimen (HR=0.71; 0.66-0.77) and when used as a categorical variable, A MPR < 85% in the previous 180 days had an increased risk of switching to a PI based regimen (HR=22.8; 11.02-47.16).

**Table 2:** Multivariable Cox PH analyses of factors associated with time to switch to a second line regimen among antiretroviral-naive patients starting Initial ART Regimens at Hospital Nacional Cayetano Heredia in Lima, Peru; January 2, 2006-December 30, 2010*.

Variable	MPR		LMC		CVC	
	AHR	95% CI	AHR	95% CI	AHR	95% CI
Age (per 10 year increase)	0.83	0.62-1.11	0.8	0.61-1.05	0.81	0.62-1.07
Sex: Female	0.87	0.45-1.68	0.8	0.41-1.54	0.78	0.40-1.52
Baseline weight < 60 Kg	1.53	0.86-2.75	1.5	0.83-2.71	1.54	0.85-2.78
Baseline CD4 (per 50 cells/ml increase)	0.88	0.76-1.04	0.94	0.82-1.08	0.95	0.83-1.09
Extreme poverty	1.6	0.79-3.23	1.30	0.65-2.6	1.33	0.67-2.65
Prior Opportunistic infection	1.37	0.6-3.11	1.18	0.53-2.59	1.12	0.5-2.47
Prior TB infection	0.81	0.43-1.54	0.94	0.5-1.75	0.94	0.5-1.76
Number of ARVs used during the first line period (4-6)^	0.73	0.31-1.75	0.57	0.24-1.36	0.61	0.26-1.45
MPR (per 10% increase)	**0.66**	**0.61-0.71**	-	-	-	-
LMC (per 10% increase)	-	-	**0.83**	**0.71-0.96**	-	-
CVC (per 10% increase)	-	-	-	-	0.91	0.79-1.05

Abbreviations: CI, confidence interval; AHR, adjusted hazard ratio MPR possession ratio; LMC, laboratory monitoring constancy; CVC, clinic visit constancy; TB, tuberculosis; ARV, antiretroviral.

^ Zidovudine and stavudine are considered as the same ARV given their similar resistance patterns.

* 747 patients were included in these analyses.

In the second model, fit for LMC, only this OP (HR=0.83; 0.71-0.96) was associated with prolonged first-line regimen persistence while other demographic, clinical and laboratory characteristics were not associated with increased first-line longevity in this model. In the third model, CVC showed a trend towards longer first-line regimens (HR=0.91; 0.79-1.05) but failed to reach statistical significance. No other demographic, clinical or laboratory variables in this model were associated with this outcome.

### Analysis of time to second-line virologic failure

We included 79 individuals switching to a PI including regimen in the analyses of factors associated with second-line regimens virologic failure (See table 1 for baseline characteristics). In UV analyses, only MPR (HR=0.89; 0.80-0.99) reached a statistically significant association with prolonged time to virologic failure in these regimens, other two variables: history of opportunistic infections (HR=0.35; 0.15-0.83) and STDs (HR=0.35; 0.13-0.92) were also associated with prolonged time to virologic failure of the second-line regimen, but we suspected these were not clinically significant and we did not include them in subsequent analyses.

We again fit three separate multivariable Cox PH models, one including each OP (Table 3). In the model that included MPR, higher MPR values (per 10%) were associated with a decreased hazard of second-line regimen virologic failure (HR=0.88; 0.77-0.99) while no demographic or clinical variables were associated with time to second-line virologic failure. The models fit for LMC and CVC failed to show any statistically significant associations with this outcome (Table 3).

**Table 3:** Multivariable Cox PH analyses of factors associated with time until virologic failure on a second line regimen at Hospital Nacional Cayetano Heredia in Lima, Peru; January 2, 2006-December 30, 2010*.

Variable	MPR		LMC		CVC	
	AHR	95% CI	AHR	95% CI	AHR	95% CI
Age (per 10 year increase)	0.74	0.47-1.18	0.70	0.44-1.1	0.72	0.44-1.16
Sex: Females	1.52	0.67-3.46	1.48	0.66-3.32	1.54	0.68-3.5
Number of new ARVs used during the second line period (2 or 3)^	0.63	0.30-1.33	0.67	0.31-1.45	0.65	0.3-1.39
Time on a first line regimen	1.02	0.98-1.06	1.01	0.97-1.05	1.01	0.97-1.05
MPR (per 10% increase)	**0.88**	**0.77-0.99**	-	-	-	-
LMC (per 10% increase)	-	-	0.92	0.77-1.09	-	-
CVC (per 10% increase)	-	-	-	-	0.96	0.81-1.13

Abbreviations: CI, confidence interval; AHR, adjusted hazard ratio; MPR, medication possession ratio; LMC, laboratory monitoring constancy; CVC, clinic visit constancy; ARV, antiretroviral.

^ Zidovudine and stavudine are considered as the same ARV given their similar resistance patterns.

* 79 patients were included in these analyses.

## Discussion

In our study, MPR measured during first-line regimens, predicted prolonged persistence of these regimens and its prognostic value endured beyond first-line therapy and was associated with time to virologic failure of second-line regimens. Laboratory monitoring constancy predicted first-line persistence but showed no association with time to second-line regimens virologic failure while CVC was not associated with either study outcome. Data collection for OPs was feasible in Peru in the context of routine care provision. The use of such OPs, in particular MPR, could be helpful to treatment programs in developing nations providing tools to identify patients at higher risk of needing a switch to more expensive second-line regimens and help identify those individuals subsequently at risk of failing those regimens. Such early warning data can be used to target resource allocation to high-risk individuals who are at need for additional interventions to ensure treatment success.

Medication possession ratio has been associated with a broad range of clinical outcomes such as virologic and immunologic treatment response [11], mortality [10], loss to follow up [20] and development of HIV drug resistance [7] both in resource-limited and rich settings. This study adds to the extant literature by investigating its impact on first-line regimen persistence and second-line regimen virologic failure in RLS. We found that MPR was able to predict ART regimen persistence in multiple incarnations in our analyses (continuously over the duration of first-line, and when measured over the preceding 180 days at any given time during first-line regimens either continuously or categorically). McMahon reported that MPR may better predict clinical outcomes when having a longer measured period and when measured closer to the studied outcome [15]; these observations applied to our study as well with better protection with increasing MPRs when measured over the total duration of first-line rather than over the preceding 180 days. We posit that individual treatment programs may select a MPR measure (i.e. continuous or categorical <85% over the first 180 days or even over longer periods) depending on their data abstraction and calculation capabilities and routinely highlighting patients with high-risk measures for additional intervention. Data collection on medication pick up was already underway as a routine part of the HIV treatment program at HNCH as part of normal clinic operations, and with minimal effort, we were able to extract these data and calculate MPR values. The use of such data in RLS not just for programmatic objectives but also for the prediction of patients at risk for sub-optimal therapeutic outcomes represent a commonly missed opportunity to favorably impact patient care at the individual level in national treatment programs.

Laboratory monitoring constancy is an underutilized tool for measuring adherence to care [16,21]. This measure has the advantage of being objectively recorded in most treatment programs with access to a laboratory, although it may be unavailable in programs with very limited resources in which HIV viral load and CD4 measurements do not occur routinely. In our study, high LMC was associated with greater persistence for first-line regimens and represents a feasible constancy measure that could be implemented as an early predictor of patients at risk for early switch to second-line regimens in treatment programs in most RLS. This measure may prove especially useful in RLS with incomplete or unavailable pharmacy and/or clinic visit records.

Clinic visit constancy has mainly been studied in the developed world [4,22], having a proven impact on outcomes such as survival [17] and viral suppression [23], but has seldom been used in developing countries as a measure of retention in care. In our study, CVC failed to prove an association with either study outcome. Currently, clinic retention is not a routinely reported outcome in Peru and the available data may be less accurately captured when compared to pharmacy-based measures, for which a reporting mandate exists as these data are used for decision making at the programmatic level. Also, given the increasing numbers of patients in need of attention and the limited number of providers, patient visits may occur at intervals greater than 6 months apart. Thus, the optimal interval for calculating CVC is not clear. A further difficulty in the systematic capture of visit adherence data is the lack of robust scheduling management systems, which would regularly capture these data as part of routine care. Lastly, CVC has been most extensively tested in developed countries where all interventions (medication pick up, laboratory draws and provider visits) occurs on the same date; in such settings, clinic visit adherence measures likely reflect a combination of medication pick-up, laboratory and clinic visit adherence as opposed to RLS like ours where these processes occur on independent dates. Future studies of CVC and other retention measures utilized in resource-rich settings in RLS should carefully consider the potential impact of these differences.

Several patient characteristics have been associated with decreased regimen persistence in the extant literature [5,16-18], among them, indirect markers of advanced HIV at ART initiation (high viral loads, low CD4 counts, low weight) [24,25], non-adherence risk factors (younger age, depression, substance abuse) [25,26] and barriers to access to care (lack of medical coverage, incarceration, female gender, MSM, racial/ethnic minorities) [24,27]. Our analyses included many of these factors and older age, male gender and those with higher weights had a trend towards better regimen persistence although they did not attain statistical significance. These differences may reflect a limitation of sample size or an indication that these variables may not be as predictive as the OPs tested in our treatment setting.

Our findings should be interpreted in the context of our limitations. We report the experience of one center in Peru and our findings may not be generalizable to other settings. Our sample size could have prevented us from detecting existing associations a larger dataset would be able to. Unmeasured patient, treatment and administrative factors may affect persistence on a first-line regimen. Lastly, we measured second-line virologic failure at 90 days rather than the more commonly used 180 days. As with all observational studies, we are able to identify associations but could not attribute causality, and unmeasured confounding may have affected our findings.

Despite our limitations, our study advances the knowledge of HIV regimen persistence in several ways. We demonstrated the feasibility of constructing OPs with data currently available in a RLS and we present a head to head comparison among three OPs regarding their predictive value of initial ART regimen persistence.

In conclusion, OPs such as MPR were associated with first-line regimen persistence and time to second-line regimen virologic failure in Peru. We propose that the use of operational parameters derived from routinely captured programmatic data hold the potential for enhancing patient care by detecting individuals at risk for abbreviated first or second-line regimen persistency. While the nature of interventions to categorize and intervene in at risk patients will need to be tailored to different populations and environments, the tactical use of data captured routinely for programmatic reporting purposes can favorably impact individual care.
